# Crystal structure of *catena*-poly[[[aqua­bis­(di­methyl­formamide-κ*O*)magnesium(II)]-μ_3_-(2,2′-bi­pyridine-5,5′-di­carboxyl­ato-κ^5^ *O* ^2^:*O* ^2′^:*N*,*N*′:*O* ^5^)-[di­chlorido­platinum(II)]] di­methyl­formamide monosolvate]

**DOI:** 10.1107/S2056989017008118

**Published:** 2017-06-07

**Authors:** Fredrik Lundvall, Mats Tilset

**Affiliations:** a Centre for Materials Science and Nanotechnology, Department of Chemistry, University of Oslo, PO Box 1126, 0315 Oslo, Norway; b Department of Chemistry, University of Oslo, PO Box 1033, 0315 Oslo, Norway

**Keywords:** crystal structure, coordination polymer, bimetallic compound, metal–organic framework, catalysis

## Abstract

The title compound, {[MgPtCl_2_(C_12_H_6_N_2_O_4_)(C_3_H_7_NO)_2_(H_2_O)]·C_3_H_7_NO}_*n*_, is a one-dimensional coordination polymer. The structure consists of Pt-functionalized bi­pyridine ligands connected by Mg^II^ cations, as well as coordinating and non-coordinating solvent mol­ecules. The Pt^II^ cation is coordinated by the two N atoms of the bi­pyridine moiety and two Cl atoms in a square-planar fashion. This coordination induces an in-plane bend along the bi­pyridine backbone of approximately 10° from the linear ideal of a conjugated π-system. Likewise, the coordination to the Mg^II^ cation induces a significant bowing of the plane of the bi­pyridine of about 12°, giving it a distinct curved appearance. The carboxyl­ate groups of the bi­pyridine ligand exhibit moderate rotations relative to their parent pyridine rings. The Mg^II^ cation has a fairly regular octa­hedral coordination polyhedron, in which three vertices are occupied by O atoms from the carboxyl­ate groups of three different bi­pyridine ligands. The remaining three vertices are occupied by the O atoms of two di­methyl­formamide (DMF) mol­ecules and one water mol­ecule. The one-dimensional chains are oriented in the [01-1] direction, and non-coordinating DMF mol­ecules can be found in the space between the chains. The shortest inter­molecular O⋯H contacts are 2.844 (4) and 2.659 (4) Å, suggesting moderate hydrogen-bonding inter­actions. In addition, there is a short inter­molecular Pt⋯Pt contact of 3.491 (1) Å, indicating a Pt stacking inter­action. Some structure-directing contribution from the hydrogen bonding and Pt⋯Pt inter­action is probable. However, the crystal packing seems to be directed primarily by van der Waals inter­actions.

## Abstract 2

A new one-dimensional coordination polymer formed unexpectedly during the synthesis of a Pt-functionalized bi­pyridine linker for metal–organic frameworks. We report here the synthesis, structure determination and energy-dispersive X-ray spectroscopy analysis of this new coordination polymer.

## Chemical context   

Metal–organic frameworks (MOFs) are porous materials that have attracted significant attention over the last two decades. The materials are formed from inorganic and organic components, typically a cationic unit linked by an organic ligand commonly referred to as a linker. Incorporating a catalytically active site in the linker of a porous MOF has the potential to create a heterogenous catalyst with the same selectivity often associated with homogenous catalysts. To this end, there are two main strategies for incorporating the active species. One method is to add the active species to the MOF after the frameworks has been formed, so called post-synthetic modification. The other option is to functionalize the linker either before or during the MOF synthesis (Cohen, 2017 ▸).

The title compound is an unexpected byproduct from the synthesis of the functionalized linker (2,2′-bi­pyridine-5,5′-dicarboxcylic acid)tetra­chlorido­platinum(IV). 2,2′-bi­pyridine-5,5′-dicarboxcylic acid is highly suitable for incorporation in the UiO-67 MOF, where it can partially substitute the biphenyl linker of the parent structure (Cavka *et al.*, 2008 ▸). Furthermore, the N atoms of the bi­pyridine linker can be used to anchor and functionalize the linker with *e.g.* Pt or other noble metals. The Pt site of the target linker is inter­esting in a catalytic context. Pt has a rich redox chemistry and is know to readily switch between oxidation states Pt^II^ and Pt^IV^, thus providing an active site for *e.g.* C—H activation. The target linker and its successful inclusion in the UiO-67 MOF has been reported in the literature (Øien *et al.*, 2015 ▸).
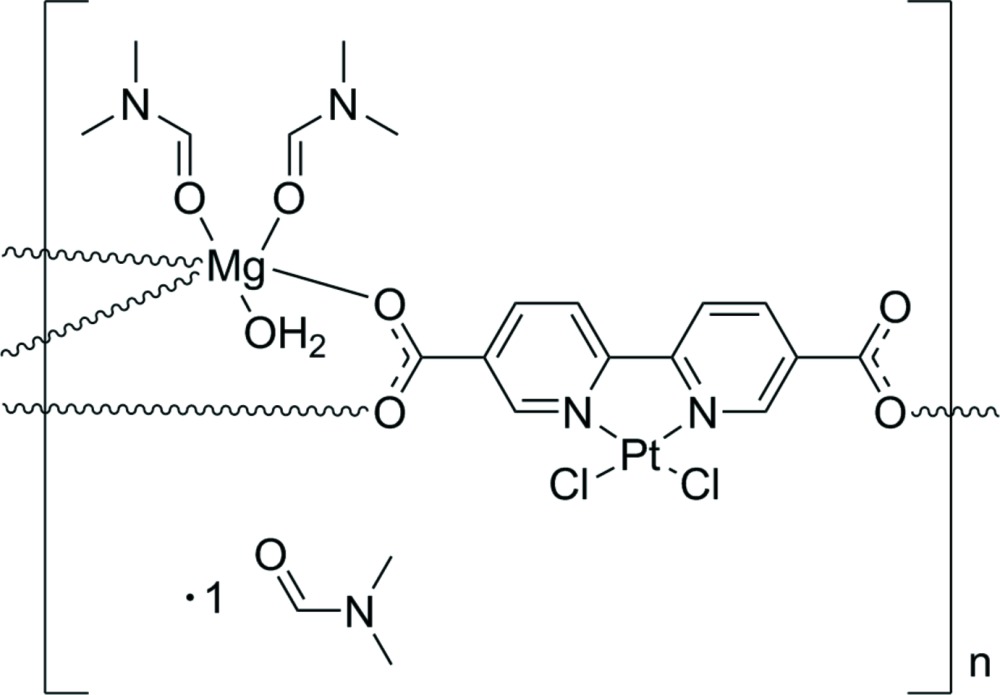


## Structural commentary   

**Figure 1:**
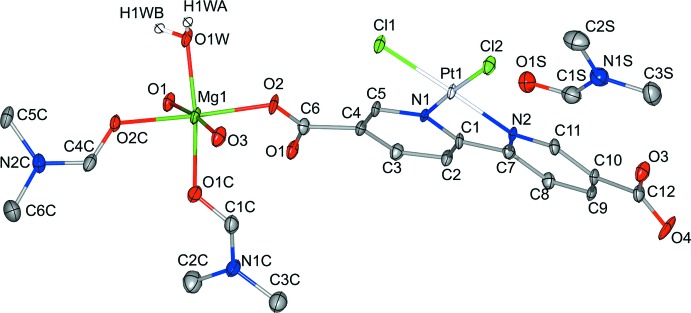
The asymmetric unit of the title compound, with atom labels and 50% probability displacement ellipsoids. H atoms have been omitted for clarity, excluding the H atoms of the coordinating water mol­ecule (H1*WA*/*B*).

The asymmetric unit of the title compound comprises a Mg^II^ cation coordinated by two di­methyl­formamide (DMF) mol­ecules and one water mol­ecule, as well as a bi­pyridine moiety with two Cl atoms and Pt^II^ in a square-planar coordination. In addition, the asymmetric unit contains a DMF solvent mol­ecule that does not coordinate to the rest of the structure (Fig. 1 ▸). The Mg^II^ cation is octa­hedrally coordinated, with the vertices occupied by O atoms from two DMF mol­ecules, one water mol­ecule and three carboxyl­ate groups from three different bi­pyridine moieties.

The carboxyl­ate groups coordinate to the cation in a monodentate fashion, thus each bi­pyridine moiety coordinates to three different Mg^II^ cations. The fourth O atom of the carboxyl­ate groups (O4) is uncoordinating, and has a more pronounced displacement ellipsoid when compared to the coordinating O atoms O1, O2 and O3. Moderate torsion angles of 12.56 (29)° and 12.29 (25)° can be observed for the two carboxyl­ate groups relative to their parent pyridine rings.

One Pt^II^ and two Cl atoms are coordinated by the N atoms of the bi­pyridine ligand in a square-planar coordination. This type of coordination is commonly observed in complexes with Pt^II^ and other transition metals with a *d* ^8^ electron configuration (Krogmann, 1969 ▸). The square plane itself is regular with an r.m.s. deviation from the flat plane of only 0.013 Å. Angles of 88.94 (5) and 80.50 (13)° are observed for Cl1—Pt1—Cl2 and N1—Pt1—N2, respectively. Notably, the Pt—Cl bonds are slightly longer (∼2.30 Å) than the Pt—N bonds (∼2.02 Å). This indicates that there is a stronger *trans* effect from the bi­pyridine ligand than the Cl atoms. The bond lengths and angles (Table 1 ▸) are consistent with other similar structures (Hazell *et al.*, 1986 ▸; Kato & Ikemori, 2003 ▸; Kato *et al.*, 2006 ▸; Hazell, 2004 ▸; Maheshwari *et al.*, 2007 ▸).

**Figure 2:**
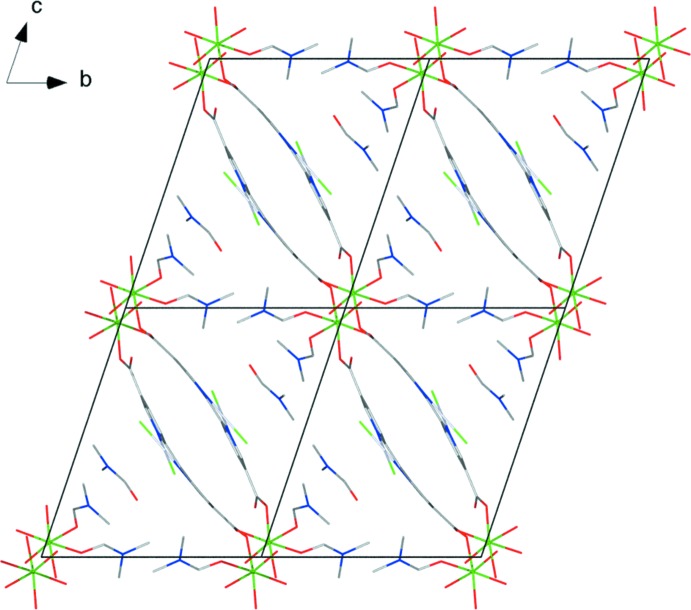
Packing diagram of the title compound, viewed along the *a* axis. H atoms have been omitted for clarity.

**Figure 4:**
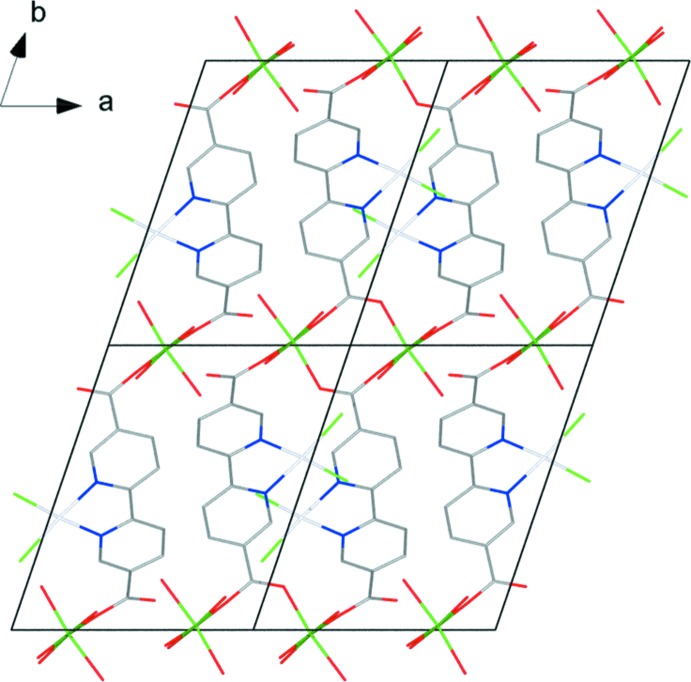
Packing diagram of the title compound, viewed along the *c* axis. H atoms, non-coordinating solvent mol­ecules and non-O atoms of coordinating solvent mol­ecules have been omitted for clarity.

The bi­pyridine backbone exhibits a distinct bowing relative to the plane of the mol­ecule (Figs. 2 ▸ and 3 ▸) as well as an in-plane bend (Fig. 4 ▸). The bowing has been calculated to 12.74 (20)° by comparing the angle between the least-squares planes of the pyridine rings. Deviations from the ideal 120° for the N1—C1—C7 and C1—C7—N2 angles give an estimation of the in-plane bending of about 10°. Such in-plane bending and bowing has been observed in several similar, albeit non-coordinating, bi­pyridine compounds (Hazell *et al.*, 1986 ▸; Kato & Ikemori, 2003 ▸; Kato *et al.*, 2006 ▸; Hazell, 2004 ▸; Maheshwari *et al.*, 2007 ▸). However, it is likely that the distortion of the bi­pyridine is influenced by the coordination to Mg as well as the inter­molecular Pt⋯Pt inter­action.

## Supra­molecular features   

The title compound forms one-dimensional chains comprising two bi­pyridine linkers and two Mg^II^ cations with associated coordinating solvent mol­ecules as the repeating unit. These chains are oriented in the [01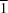] direction (Fig. 1 ▸). DMF solvent mol­ecules can be found between the chains, oriented side-on to the plane of the bi­pyridine linker. Hydrogen-bonding inter­actions (Table 2 ▸) are found between the coordinating water mol­ecule O2*W* and atoms O2*C* and O4 of neighboring DMF and bi­pyridine moieties. The donor–acceptor distances are 2.844 (4) and 2.659 (4) Å, indicating moderately strong bonds. There is also a short inter­molecular Pt⋯Pt contact of 3.491 (1) Å, indicating a Pt stacking inter­action between pairs of bi­pyridine ligands in the chain. These types of stacking inter­actions are common in square-planar complexes of metals in a *d* ^8^ electronic configuration (Krogmann, 1969 ▸). The hydrogen bonding and Pt⋯Pt stacking inter­action are likely to contribute to the overall structure and crystal packing.

## Synthesis and crystallization   

2,2′-Bi­pyridine-5,5′-di­carb­oxy­lic acid, was synthesized according to literature methods (Szeto *et al.*, 2008 ▸). Di­methyl­formamide (DMF) was supplied by Sigma–Aldrich and dried before use. K_2_PtCl_6_ and 35%_wt_ HCl were used as received from Sigma–Aldrich.

The title compound was synthesized by dissolving 16.3 mg (0.067 mmol) 2,2′-bi­pyridine-5,5′-di­carb­oxy­lic acid, 65.3 mg (0.134 mmol) K_2_PtCl_6_ and three drops of 35% HCl in 4 ml of DMF. The mixture was heated in a closed glass vial in a convection oven at 323 K for 48 h, followed by 24 h at 343 K and finally 48 h at 353 K. This procedure yielded clusters of yellow needle-shaped crystals suitable for single crystal X-ray diffraction, as well as a yet unidentified red compound.

**Figure 5:**
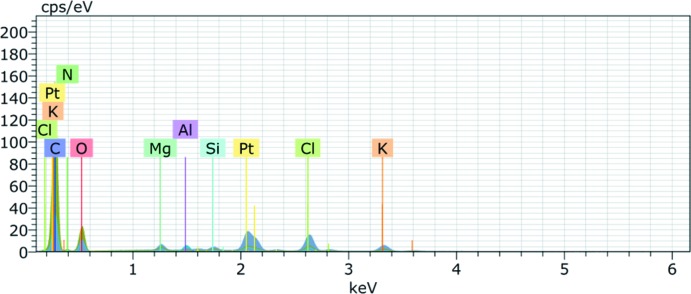
Energy-dispersive X-ray spectroscopy (EDX) spectrum of the title compound.

Note that the synthesis procedure does not include a source of Mg, despite its inclusion as cation in the title compound. The initial structural solution included K^+^ as the cation. However, the refinement of this initial model indicated several problems. First of all, a fully deprotonated organic ligand (*L* ^2−^) and just one K^+^ cation would imply a charge imbalance in the structure. Secondly, the model had unrealistic displacement ellipsoids for the metal species as well as an unusual weighting scheme. Lastly, the metal-to-oxygen bond lengths were significantly shorter than expected for K—O bonds in an octa­hedral environment when applying the bond-valence method (Brown & Altermatt, 1985 ▸). Thus we hypothesized that the coordination polymer must contain a contamination from the synthesis. The correct cation would likely be a divalent metal that is commonly encountered in organic chemistry, often exhibits octa­hedral coordination, and most importantly has a short metal-to-oxygen bond. Based on these criteria, the cation of the initial model was replaced with Mg, which solved the aforementioned refinement issues. Subsequent energy-dispersive X-ray spectroscopy (EDX) confirmed the presence of Mg in the sample (Fig. 5 ▸). The source of the contamination is likely from a batch of DMF incorrectly dried over MgSO_4_.

## Refinement   

Crystal data, data collection and structure refinement details are summarized in Table 3 ▸. H atoms were positioned geometrically at distances of 0.87 (OH), 0.95 (CH) and 0.98 Å (CH_3_) and refined using a riding model with *U*_iso_(H) = 1.2 *U*_eq_(CH) and *U*_iso_(H) = 1.5*U*_eq_(OH and CH_3_).

## Supplementary Material

Crystal structure: contains datablock(s) I. DOI: 10.1107/S2056989017008118/vn2129sup1.cif

Structure factors: contains datablock(s) I. DOI: 10.1107/S2056989017008118/vn2129Isup2.hkl

CCDC reference: 1553409

Additional supporting information:  crystallographic information; 3D view; checkCIF report

## Figures and Tables

**Figure 3:**
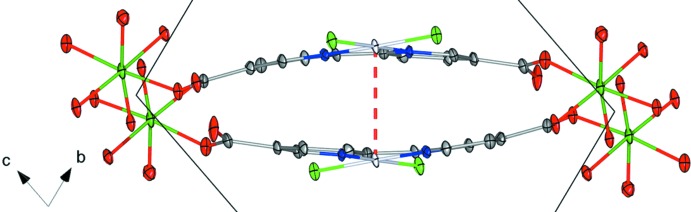
Detailed view of the title compound viewed along the *a* axis, with 50% probability displacement ellipsoids. H atoms, non-coordinating solvent mol­ecules and non-O atoms of coordinating solvent mol­ecules have been omitted for clarity. The Pt⋯Pt inter­action is indicated by a red dashed line. The second bi­pyridine moiety is generated by the symmetry operation (−*x* + 2, −*y* + 1, −*z* + 1).

**Table 1:** Selected geometric parameters (Å, °)

Pt1—Cl1	2.3000 (14)	Mg1—O2^ii^	2.066 (3)
Pt1—Cl2	2.3066 (13)	Mg1—O3^iii^	2.063 (3)
Pt1—N1	2.020 (3)	Mg1—O1*C*	2.086 (3)
Pt1—N2	2.016 (3)	Mg1—O2*C*	2.155 (3)
Pt1—Pt1^i^	3.491 (1)	Mg1—O1*W*	2.053 (3)
Mg1—O1	2.030 (3)		
			
Cl1—Pt1—Cl2	88.94 (5)	N2—Pt1—N1	80.50 (13)
N1—Pt1—Cl1	94.88 (10)	N1—C1—C7	114.8 (4)
N2—Pt1—Cl2	95.66 (10)	N2—C7—C1	114.8 (4)

Symmetry codes: (i) 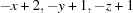; (ii) 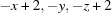; (iii) 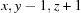.

**Table 2:** Hydrogen-bond geometry (Å, °)

*D*—H⋯*A*	*D*—H	H⋯*A*	*D*⋯*A*	*D*—H⋯*A*
O1*W*—H1*WA*⋯O2*C* ^iv^	0.87	2.05	2.844 (4)	151
O1*W*—H1*WB*⋯O4^v^	0.87	1.80	2.659 (4)	168

Symmetry codes: (iv) 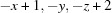; (v) 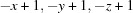.

**Table 3:** Experimental details

Crystal data	
Chemical formula	[MgPtCl_2_(C_12_H_6_N_2_O_4_)(C_3_H_7_NO)_2_(H_2_O)]·C_3_H_7_NO
*M*_r_	769.79
Crystal system, space group	Triclinic, *P* 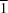
Temperature (K)	100
*a*, *b*, *c* (Å)	9.224 (4), 12.083 (6), 13.673 (7)
α, β, γ (°)	69.206 (14), 80.361 (17), 69.054 (14)
*V* (Å^3^)	1329.1 (11)
*Z*	2
Radiation type	Mo *K*α
μ (mm^−1^)	5.56
Crystal size (mm)	0.2 × 0.1 × 0.09

Data collection	
Diffractometer	Bruker PHOTON CCD
Absorption correction	Multi-scan (*SADABS*; Krause *et al.*, 2015 ▸)
*T*_min_, *T*_max_	0.518, 0.745
No. of measured, independent and observed [*I* > 2σ(*I*)] reflections	31765, 4615, 4405
*R*_int_	0.057
(sin θ/λ)_max_ (Å^−1^)	0.594

Refinement	
*R*[*F* ^2^ > 2σ(*F* ^2^)], *wR*(*F* ^2^), *S*	0.028, 0.074, 1.09
No. of reflections	4615
No. of parameters	352
H-atom treatment	H-atom parameters constrained
Δρ_max_, Δρ_min_ (e Å^−3^)	1.92, −2.42

Computer programs: *APEX3* and *SAINT* (Bruker, 2015 ▸), *SHELXT* (Sheldrick, 2015*a* ▸), *SHELXL2014* (Sheldrick, 2015*b* ▸), *OLEX2* (Dolomanov *et al.*, 2009 ▸), *DIAMOND* (Brandenburg, 2014 ▸), *ChemBioDraw Ultra* (Cambridge Soft, 2012 ▸) and *publCIF* (Westrip, 2010 ▸).

## supplementary crystallographic information

### Crystal data

**Table:**

[MgPtCl_2_(C_12_H_6_N_2_O_4_)(C_3_H_7_NO)_2_(H2O)]·C_3_H_7_NO	*Z* = 2
*M**_r_* = 769.79	*F*(000) = 756
Triclinic, *P*1	*D*_x_ = 1.923 Mg m^−^^3^
*a* = 9.224 (4) Å	Mo *K*α radiation, λ = 0.71073 Å
*b* = 12.083 (6) Å	Cell parameters from 9986 reflections
*c* = 13.673 (7) Å	θ = 2.4–24.8°
α = 69.206 (14)°	µ = 5.56 mm^−^^1^
β = 80.361 (17)°	*T* = 100 K
γ = 69.054 (14)°	Needle, clear yellow
*V* = 1329.1 (11) Å^3^	0.2 × 0.1 × 0.09 mm

### Data collection

**Table:**

Bruker PHOTON CCD diffractometer	4615 independent reflections
Radiation source: fine-focus sealed tube	4405 reflections with *I* > 2σ(*I*)
Graphite monochromator	*R*_int_ = 0.057
ω scans	θ_max_ = 25.0°, θ_min_ = 2.4°
Absorption correction: multi-scan (SADABS; Krause *et al.*, 2015)	*h* = −10→10
*T*_min_ = 0.518, *T*_max_ = 0.745	*k* = −14→14
31765 measured reflections	*l* = −16→16

### Refinement

**Table:**

Refinement on *F*^2^	Primary atom site location: structure-invariant direct methods
Least-squares matrix: full	Hydrogen site location: mixed
*R*[*F*^2^ > 2σ(*F*^2^)] = 0.028	H-atom parameters constrained
*wR*(*F*^2^) = 0.074	*w* = 1/[σ^2^(*F*_o_^2^) + (0.0491*P*)^2^ + 1.4382*P*] where *P* = (*F*_o_^2^ + 2*F*_c_^2^)/3
*S* = 1.09	(Δ/σ)_max_ = 0.002
4615 reflections	Δρ_max_ = 1.92 e Å^−^^3^
352 parameters	Δρ_min_ = −2.42 e Å^−^^3^
0 restraints	

### Special details

**Table:**

Geometry. All esds (except the esd in the dihedral angle between two l.s. planes) are estimated using the full covariance matrix. The cell esds are taken into account individually in the estimation of esds in distances, angles and torsion angles; correlations between esds in cell parameters are only used when they are defined by crystal symmetry. An approximate (isotropic) treatment of cell esds is used for estimating esds involving l.s. planes.

### Fractional atomic coordinates and isotropic or equivalent isotropic displacement parameters (Å^2^)

**Table:**

	*x*	*y*	*z*	*U*_iso_*/*U*_eq_	
Pt1	0.96005 (2)	0.60542 (2)	0.56892 (2)	0.01206 (8)	
Cl1	1.17780 (12)	0.52245 (9)	0.66544 (8)	0.0179 (2)	
Cl2	1.06049 (12)	0.75785 (9)	0.45784 (8)	0.0178 (2)	
Mg1	0.75731 (15)	0.01424 (12)	1.05885 (10)	0.0146 (3)	
O1	0.8504 (3)	0.0973 (3)	0.9186 (2)	0.0170 (6)	
O2	1.0637 (3)	0.1526 (3)	0.8926 (2)	0.0191 (6)	
O3	0.6600 (4)	0.9454 (3)	0.2053 (2)	0.0210 (7)	
O4	0.4507 (4)	0.8962 (3)	0.1999 (2)	0.0305 (8)	
O1C	0.8374 (3)	0.1152 (3)	1.1211 (2)	0.0205 (6)	
O2C	0.5514 (3)	0.1750 (3)	1.0171 (2)	0.0172 (6)	
O1W	0.6744 (3)	−0.0626 (3)	0.9774 (2)	0.0167 (6)	
H1WA	0.6049	−0.1000	1.0028	0.025*	
H1WB	0.6456	−0.0055	0.9174	0.025*	
N1	0.8564 (4)	0.4808 (3)	0.6630 (3)	0.0105 (7)	
N2	0.7619 (4)	0.6682 (3)	0.4934 (3)	0.0117 (7)	
N1C	1.0118 (4)	0.1352 (4)	1.2069 (3)	0.0226 (8)	
N2C	0.3786 (4)	0.3657 (3)	1.0119 (3)	0.0244 (8)	
C1	0.7083 (5)	0.5059 (4)	0.6397 (3)	0.0137 (8)	
C2	0.6205 (5)	0.4317 (4)	0.6998 (3)	0.0146 (8)	
H2	0.5144	0.4540	0.6859	0.017*	
C3	0.6879 (5)	0.3246 (4)	0.7803 (3)	0.0165 (9)	
H3	0.6298	0.2717	0.8215	0.020*	
C4	0.8419 (5)	0.2961 (4)	0.7994 (3)	0.0137 (8)	
C5	0.9213 (5)	0.3777 (4)	0.7419 (3)	0.0147 (8)	
H5	1.0247	0.3606	0.7586	0.018*	
C6	0.9253 (5)	0.1716 (4)	0.8792 (3)	0.0147 (8)	
C7	0.6553 (5)	0.6113 (4)	0.5438 (3)	0.0133 (8)	
C8	0.5117 (5)	0.6468 (4)	0.5021 (3)	0.0155 (9)	
H8	0.4370	0.6076	0.5391	0.019*	
C9	0.4788 (5)	0.7396 (4)	0.4065 (3)	0.0151 (8)	
H9	0.3803	0.7661	0.3778	0.018*	
C10	0.5910 (5)	0.7938 (4)	0.3526 (3)	0.0144 (8)	
C11	0.7305 (5)	0.7576 (3)	0.3990 (3)	0.0127 (8)	
H11	0.8060	0.7966	0.3634	0.015*	
C12	0.5650 (5)	0.8877 (4)	0.2425 (3)	0.0168 (9)	
C1C	0.9292 (5)	0.0737 (4)	1.1917 (3)	0.0207 (9)	
H1C	0.9411	−0.0084	1.2386	0.025*	
C2C	1.0120 (6)	0.2562 (5)	1.1336 (4)	0.0316 (11)	
H2CA	0.9431	0.2807	1.0769	0.047*	
H2CB	1.1178	0.2512	1.1044	0.047*	
H2CC	0.9753	0.3186	1.1702	0.047*	
C3C	1.1173 (6)	0.0797 (5)	1.2919 (4)	0.0294 (11)	
H3CA	1.2248	0.0591	1.2634	0.044*	
H3CB	1.0986	0.0033	1.3402	0.044*	
H3CC	1.0997	0.1393	1.3295	0.044*	
C4C	0.4982 (5)	0.2652 (4)	1.0503 (3)	0.0212 (9)	
H4C	0.5468	0.2621	1.1077	0.025*	
C5C	0.3018 (5)	0.3840 (4)	0.9197 (4)	0.0250 (10)	
H5CA	0.3560	0.3146	0.8919	0.038*	
H5CB	0.3041	0.4629	0.8661	0.038*	
H5CC	0.1937	0.3867	0.9391	0.038*	
C6C	0.3190 (7)	0.4667 (5)	1.0579 (5)	0.0389 (14)	
H6CA	0.2148	0.4698	1.0898	0.058*	
H6CB	0.3138	0.5465	1.0031	0.058*	
H6CC	0.3884	0.4514	1.1116	0.058*	
O1S	0.3963 (4)	0.6539 (3)	0.7698 (3)	0.0312 (8)	
N1S	0.3595 (5)	0.8390 (4)	0.6353 (3)	0.0270 (9)	
C1S	0.3216 (6)	0.7374 (4)	0.6968 (4)	0.0266 (10)	
H1S	0.2290	0.7298	0.6826	0.032*	
C2S	0.5037 (7)	0.8550 (6)	0.6488 (5)	0.0377 (14)	
H2SA	0.5607	0.8737	0.5810	0.056*	
H2SB	0.5677	0.7779	0.6977	0.056*	
H2SC	0.4795	0.9242	0.6768	0.056*	
C3S	0.2617 (6)	0.9344 (5)	0.5520 (4)	0.0344 (12)	
H3SA	0.2239	1.0142	0.5664	0.052*	
H3SB	0.1731	0.9096	0.5482	0.052*	
H3SC	0.3222	0.9438	0.4851	0.052*	

### Atomic displacement parameters (Å^2^)

**Table:**

	*U*^11^	*U*^22^	*U*^33^	*U*^12^	*U*^13^	*U*^23^
Pt1	0.01192 (11)	0.00874 (11)	0.01110 (11)	−0.00185 (7)	−0.00702 (7)	0.00337 (7)
Cl1	0.0156 (5)	0.0152 (5)	0.0185 (5)	−0.0041 (4)	−0.0110 (4)	0.0037 (4)
Cl2	0.0181 (5)	0.0143 (5)	0.0167 (5)	−0.0071 (4)	−0.0068 (4)	0.0047 (4)
Mg1	0.0143 (7)	0.0117 (7)	0.0126 (7)	−0.0027 (6)	−0.0079 (5)	0.0040 (5)
O1	0.0173 (15)	0.0150 (15)	0.0134 (15)	−0.0046 (13)	−0.0072 (12)	0.0039 (12)
O2	0.0138 (15)	0.0148 (15)	0.0200 (16)	−0.0026 (12)	−0.0116 (12)	0.0072 (12)
O3	0.0221 (17)	0.0205 (16)	0.0153 (15)	−0.0075 (14)	−0.0065 (13)	0.0032 (12)
O4	0.0327 (19)	0.0318 (19)	0.0207 (17)	−0.0173 (15)	−0.0225 (14)	0.0153 (14)
O1C	0.0206 (16)	0.0189 (15)	0.0194 (16)	−0.0039 (13)	−0.0095 (13)	−0.0018 (13)
O2C	0.0181 (15)	0.0121 (14)	0.0163 (15)	−0.0016 (12)	−0.0087 (12)	0.0018 (12)
O1W	0.0152 (15)	0.0135 (15)	0.0148 (15)	−0.0029 (12)	−0.0098 (12)	0.0052 (11)
N1	0.0101 (17)	0.0087 (16)	0.0101 (16)	−0.0001 (14)	−0.0071 (13)	0.0000 (13)
N2	0.0133 (17)	0.0097 (17)	0.0079 (16)	−0.0008 (14)	−0.0055 (13)	0.0012 (13)
N1C	0.025 (2)	0.019 (2)	0.022 (2)	−0.0069 (17)	−0.0112 (16)	−0.0008 (16)
N2C	0.024 (2)	0.0141 (19)	0.029 (2)	0.0016 (16)	−0.0122 (17)	−0.0020 (16)
C1	0.015 (2)	0.0081 (19)	0.012 (2)	0.0018 (16)	−0.0072 (16)	0.0007 (15)
C2	0.013 (2)	0.013 (2)	0.014 (2)	−0.0027 (17)	−0.0063 (16)	0.0014 (16)
C3	0.019 (2)	0.015 (2)	0.013 (2)	−0.0057 (18)	−0.0029 (17)	0.0005 (16)
C4	0.015 (2)	0.010 (2)	0.011 (2)	−0.0001 (17)	−0.0069 (16)	0.0009 (16)
C5	0.017 (2)	0.012 (2)	0.011 (2)	0.0008 (17)	−0.0096 (16)	0.0008 (16)
C6	0.017 (2)	0.012 (2)	0.011 (2)	−0.0039 (17)	−0.0033 (16)	0.0011 (16)
C7	0.019 (2)	0.0083 (19)	0.0097 (19)	−0.0027 (17)	−0.0033 (16)	−0.0001 (15)
C8	0.013 (2)	0.013 (2)	0.017 (2)	−0.0034 (17)	−0.0025 (17)	−0.0009 (17)
C9	0.016 (2)	0.0121 (19)	0.012 (2)	−0.0011 (17)	−0.0098 (16)	0.0020 (16)
C10	0.017 (2)	0.0095 (19)	0.012 (2)	−0.0010 (16)	−0.0072 (16)	0.0021 (15)
C11	0.014 (2)	0.0069 (18)	0.013 (2)	−0.0013 (16)	−0.0037 (16)	0.0011 (15)
C12	0.018 (2)	0.011 (2)	0.016 (2)	−0.0021 (18)	−0.0054 (17)	0.0023 (17)
C1C	0.023 (2)	0.015 (2)	0.019 (2)	−0.0009 (18)	−0.0063 (19)	−0.0012 (18)
C2C	0.031 (3)	0.024 (3)	0.036 (3)	−0.012 (2)	−0.007 (2)	−0.001 (2)
C3C	0.028 (3)	0.027 (3)	0.032 (3)	−0.008 (2)	−0.010 (2)	−0.005 (2)
C4C	0.021 (2)	0.021 (2)	0.019 (2)	−0.0062 (19)	−0.0105 (18)	0.0002 (18)
C5C	0.023 (2)	0.022 (2)	0.021 (2)	−0.001 (2)	−0.0126 (19)	0.0025 (19)
C6C	0.040 (3)	0.025 (3)	0.047 (3)	0.006 (2)	−0.020 (3)	−0.016 (2)
O1S	0.037 (2)	0.0217 (17)	0.0297 (19)	−0.0040 (15)	−0.0119 (15)	−0.0030 (15)
N1S	0.029 (2)	0.022 (2)	0.029 (2)	−0.0078 (18)	−0.0028 (18)	−0.0077 (17)
C1S	0.030 (3)	0.024 (2)	0.028 (3)	−0.008 (2)	−0.002 (2)	−0.011 (2)
C2S	0.036 (3)	0.039 (3)	0.048 (4)	−0.016 (3)	0.002 (3)	−0.023 (3)
C3S	0.042 (3)	0.022 (3)	0.030 (3)	−0.004 (2)	−0.005 (2)	−0.003 (2)

### Geometric parameters (Å, º)

**Table:**

Pt1—Cl1	2.3000 (14)	C4—C5	1.378 (6)
Pt1—Cl2	2.3066 (13)	C4—C6	1.527 (5)
Pt1—N1	2.020 (3)	C5—H5	0.9500
Pt1—N2	2.016 (3)	C7—C8	1.392 (6)
Pt1—Pt1^i^	3.491 (1)	C8—H8	0.9500
Mg1—O1	2.030 (3)	C8—C9	1.381 (6)
Mg1—O2^ii^	2.066 (3)	C9—H9	0.9500
Mg1—O3^iii^	2.063 (3)	C9—C10	1.390 (6)
Mg1—O1C	2.086 (3)	C10—C11	1.386 (6)
Mg1—O2C	2.155 (3)	C10—C12	1.525 (6)
Mg1—O1W	2.053 (3)	C11—H11	0.9500
O1—C6	1.245 (5)	C1C—H1C	0.9500
O2—Mg1^ii^	2.066 (3)	C2C—H2CA	0.9800
O2—C6	1.248 (5)	C2C—H2CB	0.9800
O3—Mg1^iv^	2.063 (3)	C2C—H2CC	0.9800
O3—C12	1.245 (5)	C3C—H3CA	0.9800
O4—C12	1.244 (5)	C3C—H3CB	0.9800
O1C—C1C	1.236 (5)	C3C—H3CC	0.9800
O2C—C4C	1.236 (5)	C4C—H4C	0.9500
O1W—H1WA	0.8696	C5C—H5CA	0.9800
O1W—H1WB	0.8718	C5C—H5CB	0.9800
N1—C1	1.356 (5)	C5C—H5CC	0.9800
N1—C5	1.343 (5)	C6C—H6CA	0.9800
N2—C7	1.354 (5)	C6C—H6CB	0.9800
N2—C11	1.352 (5)	C6C—H6CC	0.9800
N1C—C1C	1.319 (6)	O1S—C1S	1.227 (6)
N1C—C2C	1.451 (6)	N1S—C1S	1.346 (6)
N1C—C3C	1.452 (6)	N1S—C2S	1.461 (7)
N2C—C4C	1.324 (6)	N1S—C3S	1.452 (6)
N2C—C5C	1.460 (6)	C1S—H1S	0.9500
N2C—C6C	1.461 (6)	C2S—H2SA	0.9800
C1—C2	1.382 (6)	C2S—H2SB	0.9800
C1—C7	1.472 (5)	C2S—H2SC	0.9800
C2—H2	0.9500	C3S—H3SA	0.9800
C2—C3	1.385 (6)	C3S—H3SB	0.9800
C3—H3	0.9500	C3S—H3SC	0.9800
C3—C4	1.384 (6)		
			
Cl1—Pt1—Cl2	88.94 (5)	C9—C8—C7	119.4 (4)
N1—Pt1—Cl1	94.88 (10)	C9—C8—H8	120.3
N1—Pt1—Cl2	175.78 (9)	C8—C9—H9	120.3
N2—Pt1—Cl1	175.36 (9)	C8—C9—C10	119.3 (4)
N2—Pt1—Cl2	95.66 (10)	C10—C9—H9	120.3
N2—Pt1—N1	80.50 (13)	C9—C10—C12	120.9 (4)
O1—Mg1—O2^ii^	100.05 (13)	C11—C10—C9	118.9 (4)
O1—Mg1—O3^iii^	174.30 (14)	C11—C10—C12	120.1 (4)
O1—Mg1—O1C	87.00 (13)	N2—C11—C10	121.8 (4)
O1—Mg1—O2C	85.67 (12)	N2—C11—H11	119.1
O1—Mg1—O1W	85.74 (13)	C10—C11—H11	119.1
O2^ii^—Mg1—O1C	96.54 (13)	O3—C12—C10	117.0 (4)
O2^ii^—Mg1—O2C	172.91 (13)	O4—C12—O3	127.7 (4)
O3^iii^—Mg1—O2^ii^	83.17 (13)	O4—C12—C10	115.3 (4)
O3^iii^—Mg1—O1C	87.97 (13)	O1C—C1C—N1C	124.9 (4)
O3^iii^—Mg1—O2C	91.48 (13)	O1C—C1C—H1C	117.6
O1C—Mg1—O2C	87.89 (13)	N1C—C1C—H1C	117.6
O1W—Mg1—O2^ii^	89.21 (13)	N1C—C2C—H2CA	109.5
O1W—Mg1—O3^iii^	99.05 (14)	N1C—C2C—H2CB	109.5
O1W—Mg1—O1C	171.43 (13)	N1C—C2C—H2CC	109.5
O1W—Mg1—O2C	87.02 (12)	H2CA—C2C—H2CB	109.5
C6—O1—Mg1	140.5 (3)	H2CA—C2C—H2CC	109.5
C6—O2—Mg1^ii^	129.6 (3)	H2CB—C2C—H2CC	109.5
C12—O3—Mg1^iv^	136.2 (3)	N1C—C3C—H3CA	109.5
C1C—O1C—Mg1	127.8 (3)	N1C—C3C—H3CB	109.5
C4C—O2C—Mg1	129.8 (3)	N1C—C3C—H3CC	109.5
Mg1—O1W—H1WA	124.0	H3CA—C3C—H3CB	109.5
Mg1—O1W—H1WB	107.2	H3CA—C3C—H3CC	109.5
H1WA—O1W—H1WB	109.4	H3CB—C3C—H3CC	109.5
C1—N1—Pt1	114.5 (3)	O2C—C4C—N2C	124.9 (4)
C5—N1—Pt1	126.3 (3)	O2C—C4C—H4C	117.6
C5—N1—C1	119.2 (3)	N2C—C4C—H4C	117.6
C7—N2—Pt1	114.8 (3)	N2C—C5C—H5CA	109.5
C11—N2—Pt1	125.9 (3)	N2C—C5C—H5CB	109.5
C11—N2—C7	119.3 (3)	N2C—C5C—H5CC	109.5
C1C—N1C—C2C	121.3 (4)	H5CA—C5C—H5CB	109.5
C1C—N1C—C3C	121.5 (4)	H5CA—C5C—H5CC	109.5
C2C—N1C—C3C	116.9 (4)	H5CB—C5C—H5CC	109.5
C4C—N2C—C5C	122.1 (4)	N2C—C6C—H6CA	109.5
C4C—N2C—C6C	121.6 (4)	N2C—C6C—H6CB	109.5
C5C—N2C—C6C	116.2 (4)	N2C—C6C—H6CC	109.5
N1—C1—C2	121.1 (4)	H6CA—C6C—H6CB	109.5
N1—C1—C7	114.8 (4)	H6CA—C6C—H6CC	109.5
C2—C1—C7	124.0 (4)	H6CB—C6C—H6CC	109.5
C1—C2—H2	120.2	C1S—N1S—C2S	120.6 (5)
C1—C2—C3	119.6 (4)	C1S—N1S—C3S	121.7 (4)
C3—C2—H2	120.2	C3S—N1S—C2S	117.7 (4)
C2—C3—H3	120.7	O1S—C1S—N1S	125.7 (5)
C4—C3—C2	118.6 (4)	O1S—C1S—H1S	117.1
C4—C3—H3	120.7	N1S—C1S—H1S	117.1
C3—C4—C6	120.0 (4)	N1S—C2S—H2SA	109.5
C5—C4—C3	119.5 (4)	N1S—C2S—H2SB	109.5
C5—C4—C6	120.5 (4)	N1S—C2S—H2SC	109.5
N1—C5—C4	121.8 (4)	H2SA—C2S—H2SB	109.5
N1—C5—H5	119.1	H2SA—C2S—H2SC	109.5
C4—C5—H5	119.1	H2SB—C2S—H2SC	109.5
O1—C6—O2	127.2 (4)	N1S—C3S—H3SA	109.5
O1—C6—C4	116.2 (4)	N1S—C3S—H3SB	109.5
O2—C6—C4	116.4 (3)	N1S—C3S—H3SC	109.5
N2—C7—C1	114.8 (4)	H3SA—C3S—H3SB	109.5
N2—C7—C8	121.2 (4)	H3SA—C3S—H3SC	109.5
C8—C7—C1	123.9 (4)	H3SB—C3S—H3SC	109.5
C7—C8—H8	120.3		
			
Pt1—N1—C1—C2	−177.5 (3)	C3—C4—C6—O1	4.5 (6)
Pt1—N1—C1—C7	6.2 (4)	C3—C4—C6—O2	−179.7 (4)
Pt1—N1—C5—C4	−177.8 (3)	C5—N1—C1—C2	4.0 (6)
Pt1—N2—C7—C1	−5.4 (4)	C5—N1—C1—C7	−172.3 (3)
Pt1—N2—C7—C8	178.6 (3)	C5—C4—C6—O1	−171.8 (4)
Pt1—N2—C11—C10	179.3 (3)	C5—C4—C6—O2	3.9 (6)
Mg1—O1—C6—O2	69.9 (6)	C6—C4—C5—N1	172.1 (4)
Mg1—O1—C6—C4	−115.0 (4)	C7—N2—C11—C10	0.8 (6)
Mg1^ii^—O2—C6—O1	22.0 (6)	C7—C1—C2—C3	171.1 (4)
Mg1^ii^—O2—C6—C4	−153.2 (3)	C7—C8—C9—C10	1.4 (6)
Mg1^iv^—O3—C12—O4	−33.1 (7)	C8—C9—C10—C11	−3.3 (6)
Mg1^iv^—O3—C12—C10	145.9 (3)	C8—C9—C10—C12	174.3 (4)
Mg1—O1C—C1C—N1C	158.2 (3)	C9—C10—C11—N2	2.2 (6)
Mg1—O2C—C4C—N2C	172.9 (3)	C9—C10—C12—O3	170.0 (4)
N1—C1—C2—C3	−4.8 (6)	C9—C10—C12—O4	−10.9 (6)
N1—C1—C7—N2	−0.5 (5)	C11—N2—C7—C1	173.2 (3)
N1—C1—C7—C8	175.3 (4)	C11—N2—C7—C8	−2.7 (6)
N2—C7—C8—C9	1.6 (6)	C11—C10—C12—O3	−12.4 (6)
C1—N1—C5—C4	0.6 (6)	C11—C10—C12—O4	166.7 (4)
C1—C2—C3—C4	1.1 (6)	C12—C10—C11—N2	−175.4 (4)
C1—C7—C8—C9	−174.0 (4)	C2C—N1C—C1C—O1C	−5.8 (7)
C2—C1—C7—N2	−176.8 (4)	C3C—N1C—C1C—O1C	180.0 (4)
C2—C1—C7—C8	−0.9 (6)	C5C—N2C—C4C—O2C	−4.1 (7)
C2—C3—C4—C5	3.3 (6)	C6C—N2C—C4C—O2C	177.8 (5)
C2—C3—C4—C6	−173.1 (4)	C2S—N1S—C1S—O1S	2.9 (7)
C3—C4—C5—N1	−4.2 (6)	C3S—N1S—C1S—O1S	−178.7 (4)

Symmetry codes: (i) −*x*+2, −*y*+1, −*z*+1; (ii) −*x*+2, −*y*, −*z*+2; (iii) *x*, *y*−1, *z*+1; (iv) *x*, *y*+1, *z*−1.

### Hydrogen-bond geometry (Å, º)

**Table:**

*D*—H···*A*	*D*—H	H···*A*	*D*···*A*	*D*—H···*A*
O1*W*—H1*WA*···O2*C*^v^	0.87	2.05	2.844 (4)	151
O1*W*—H1*WB*···O4^vi^	0.87	1.80	2.659 (4)	168

Symmetry codes: (v) −*x*+1, −*y*, −*z*+2; (vi) −*x*+1, −*y*+1, −*z*+1.
